# Education-based stigma and discrimination among young adults not in 4-year college

**DOI:** 10.1186/s40359-022-00737-4

**Published:** 2022-02-08

**Authors:** Matthew K. Meisel, Michelle Haikalis, Suzanne M. Colby, Nancy P. Barnett

**Affiliations:** Center for Alcohol and Addiction Studies, Department of Behavioral and Social Sciences, Brown University School of Public Health, Box G-S121-4, Providence, RI 02903 USA

**Keywords:** Stigma, Discrimination, Noncollege, Emerging adults

## Abstract

**Background:**

Lower levels of education are strongly associated with negative health outcomes. The current study examined the degree to which those without a history of 4-year college attendance experience social stigmatization of their educational status and if these experiences are associated with mental health symptoms.

**Methods:**

Data was obtained from 488 emerging adults who never attended 4-year college using Qualtrics Panels.

**Results:**

79.4% of participants agreed to one of the six statements that not attending 4-year college is stigmatized, and 71.8% endorsed experiencing at least one form of discrimination. Higher levels of education-related stigma and more frequent experiences of education-related discrimination was associated with greater past-month anxiety and depression symptoms.

**Conclusions:**

These findings could serve to increase awareness regarding the unique and significant discrimination faced by young adults who do not attend 4-year college and identify specific areas of intervention that can help these young adults cope with the effects of stigma and discrimination.

## Background

Education is uniquely and significantly associated with both physical and mental health, such that lower levels of education are related to worse health outcomes. Individuals with lower levels of education are more likely to be depressed [1] and are more likely to smoke heavily [2]. Conversely, those with higher levels of education are more likely to engage in various preventive behaviors, such as getting mammograms, flu shots, and other health tests such as colorectal screening, and are more likely to comply with HIV and diabetes treatments [3, 4]. Thus, not surprisingly, those with lower levels of education have a shorter life expectancy [5]. These differences in life expectancy by education are persistent over time [5–7] and are well-documented across several countries [8–10]. These disparities in health are tied closely to income [11] and to the skills and opportunities that people have based on their education.

Emerging adulthood, defined as the period between the ages of 18 and 25, is a significant time in life in which individuals diverge from one another in their life trajectories [12–14]. Post high school, individuals have many options, including attending a 2- or 4-year college, attending trade school, heading directly into the job market, or joining the military. Most research on emerging adulthood has focused on 4-year college students—even though, by the age of 25, only 37% of emerging adults in the US had received a bachelor’s degree or higher [15]. It is critical that research expands on improving our understanding of the lives and trajectories of young adults who do not attend college [16].

Consistent with findings in wider age groups, health disparities based on education are also documented among emerging adults. For example, American women who did not attend 4-year college were more likely to report higher levels of anger, depressed mood and social isolation, and reported lower levels of self-esteem than their college-attending counterparts [17]. In another study of French young adults, non-college emerging adults were more likely to report greater levels of psychological distress, major depressive disorder, at least one anxiety disorder, greater isolation, and lower social support [18]. One understudied possibility as to why mental health is poorer among those with lesser education is that these individuals experience stress related to social stigmatization of their educational status.

Social stigma involves negative attitudes or discrimination against someone based on some characteristic, such as gender, sexuality, or race [19]. Stigma manifests in many ways, including public stigma, stigma by association, self-stigma and structural stigma [20], that ultimately serve to socially exclude stigmatized persons and perpetuate social inequalities [21]. Meta-analytic research confirms that stigma is a significant risk factor for mental health outcomes [22]. While stigma has often been investigated on topics such as race and sexual orientation, little has been investigated on the topic of education. However, there is some evidence of education-related stigma. Using qualitative methods, in a sample of community college students transferring to 4-year college, students felt stigmatized because they attended community college and felt their parents facilitated the stigma about attending community college [23, 24]. Guided by social stigmatization theory, the current study examined 1) the extent to which young adults who do not attend 4-year college experienced stigma and discrimination based on their education and 2) if experiences of stigma and discrimination in this group were negatively associated with mental health symptoms. Findings of this study can provide insight into better understanding the association between education and health outcomes.

## Methods

### Participants and procedure

Qualtrics surveys panels were used to collect the data. Qualtrics panels are a quick and useful method to collect psychological data using national samples. Panelists are recruited from various sources, including targeted email lists, customer loyalty portals, and social media. These individuals may be airline customers who chose to join in reward for airline miles, general consumers who participant for cash or gift cards, as well as retails customers who earn points at a retail store. Qualtrics also collects some demographic data on these panelists, such as their age and race, and then invites those who may be eligible to participate in different research studies. Interested panelists completed the four-item screener and those eligible were routed to the informed consent and then the one-time online survey. Thus, the current study utilized non-probability sampling. Eligibility criteria for the study were: (1) 18—25 years old, (2) not in the process of obtaining a high school diploma, (3) never attended 4-year college, and (4) had no plans on enrolling in a 4-year college or university in the next 12 months. All data was collected in February and March of 2021. All procedures were approved by the Brown University Institutional Review Board (protocol # 2012002863). Informed consent was obtained from all participants.

A total of 513 participants completed the survey. Data was flagged for 25 participants (4.9%) due to inconsistent responding (e.g., participant stated that they dropped out of high school and did not complete a GED but then said they went to community college or trade school). Excluding these participants resulted in a sample of 488.

### Power and sample size

A meta-analysis on the association between stigma and mental health indicates an average effect size of 0.28 [22]. Using GPower [25], we were able to conclude that our sample size of 488 was sufficient to detect a medium effect size with > 80% power [26].

### Measures

#### Demographic characteristics

Age, sex assigned at birth, gender identity, race, and ethnicity were assessed. We also measured two forms of income. Personal income was measured using the following item “This next question asks about your own personal income. Your personal income includes wages and salaries, unemployment insurance, disability payments, child support payments, or other kinds of income. What was your income, before taxes, in the last calendar year?” Household income was assessed using the following item “When answering this next question, please remember to include your income PLUS the income of all family members living in this household. What is your best estimate of the total income of all family members from all sources, before taxes, in the last calendar year?”. Response options for both items were: (1) $0–$19,999, (2) $20,000–$49,999, (3) $50,000–$74,999, (4) $75,000–$99,999, (5) $100,000–$149,999, (6) $150,000–$199,999, (7) $200,000–$249,999, (8) $250,000–$299,999 and (9) $300,000 or more. Participants could also indicate “Prefer not to answer”.

#### Stigma

A six-item questionnaire related to perceived stigma was adapted from the HIV stigma scale for the purposes of the study [27]; items were revised to reflect stigma specific to not attending 4-year college. We aimed to have at least one item that captured 3 of the 4 subscales: (1) personalized stigma, (2) concerns about public attitudes, and (3) negative self-image. The subscale disclosure concerns (which referred to HIV disclosure) was not relevant for this sample so it was not included. Participants indicated their agreement with each statement on a 4-point Likert scale, ranging from 1 (*Strongly disagree*) to 4 (*Strongly agree*). Higher scores indicate greater perceived stigma. Internal consistency was strong (α = 0.81). Items are presented in Table 1.

**Table 1:** Descriptive information of the stigma and discrimination items

Stigma items	*M*	*SD*	Strongly agree	Somewhat agree	Somewhat disagree	Strongly disagree		
1. People who do not attend 4-year college are treated worse than those who do attend	2.43	1.00	15.9	33.7	28.3	22.1		
2. Most people believe that a person who does not attend 4-year college is uneducated	2.60	1.01	19.8	39.4	21.4	19.4		
3. I feel bad about myself because I did not go to 4-year college	2.14	1.07	13.2	26.0	22.7	38.1		
4. My family treated me badly because I did not go to 4-year college	1.94	1.06	11.1	19.2	21.9	47.8		
5. My friends treated me badly because I did not go to 4-year college	1.78	0.98	8.2	14.8	23.5	53.4		
6. I feel like I have less access to resources because I did not go to 4-year college	2.40	1.06	18.1	29.3	27.0	25.6		

Discrimination Items	*M*	*SD*	Almost everyday	At least once a week	A few times a month	A few times a year	Less than once a year	Never
1. You are treated with less courtesy than others	2.66	1.75	9.3	9.1	16.3	12.8	9.5	43.2
2. You are treated with less respect than others	2.78	1.74	9.9	9.5	15.3	14.0	12.6	38.8
3. People act as if they think you are not smart	3.03	1.84	13.0	12.8	18.6	11.5	8.2	35.9
4. People act as if they are better than you	3.18	1.86	15.3	14.2	16.1	14.4	7.6	32.4

Means and SDs are presented in the table as well as the percent of participants agreeing to each response option

#### Discrimination

A four-item measure of discrimination was adapted from the Everyday Discrimination Scale [28]. Four items from this nine-item measure were identified as most relevant for education-related discrimination. Items not included were “You receive poorer service than other people at restaurants or stores”, “People are afraid of you”, and “You are threatened or harassed” because we posited that these aspects of discrimination are not relevant for education-based discrimination. We also adapted the stem of the measure to ask specifically about education-related discrimination. Participants were presented with the instructions “The next set of questions are about any discrimination that you felt that you have received based on your education. In your day-to-day life how often have any of the following things happened to you, because you did not go to 4-year college?” Response options ranged from *1 (Never)* to 6 (*Almost every day)* with higher scores indicating greater levels of perceived discrimination. Internal consistency was strong (α = 0.92). Items are presented in Table 1.

#### Anxiety and depressed mood

We used the 4-item Patient Health Questionnaire [29]. Participants rated how often they have been bothered by two symptoms each of anxiety and depression in the past month on a scale ranging from 1 (*Not at all)* to *4 (Nearly every day)*. Higher scores indicate greater symptom severity. Both the anxiety (α = 0.87) and depression (α = 0.83) scales demonstrated adequate reliability.

### Data analysis

First, descriptive statistics on demographic characteristics were calculated using SPSS 26.0. An overall mean was calculated for the discrimination and stigma scales. For descriptive purpose, items were also dichotomized. For the stigma scale, a 1 was assigned if the participant *Agreed* or *Strongly agreed* to the item and a 0 if the participant *Disagreed* or *Strongly disagreed* to the item. For the discrimination scale, a 1 was assigned if the participant indicated that the item occurred at least *Once in the past year* and a 0 if the participants indicated *Never*. Next, correlations and independent samples t-tests were conducted to examine the relationship between the demographic variables and the mean values of the stigma and discrimination scales. Lastly, four separate regression analyses were conducted to examine the association between: (1) mean-level stigma and the total number of anxiety symptoms, (2) mean-level stigma and total number of depression symptoms, (3) mean-level discrimination and total number of anxiety symptoms, and (4) mean-level discrimination and total number of depression symptoms. The significance level for all analyses was 0.05.

## Results

The average age of the sample was 21.49 (*SD* = 2.18). Most of the sample identified as female (50.4%) followed by male (45.7%), gender non-conforming (1.4%), other (1.0%), trans male (1.0%), and trans female (0.4%). Racial breakdown was as follows: 78.3% White, 13.5% Black, 2.5% multiracial, 1.4% American Indian or Alaskan Native, 1.0% Asian, and 0.4% Native Hawaiian or other Pacific Islander; 2.9% were missing on this item. Hispanic ethnicity was reported by 13.1%. Regarding personal income, most participants were either in the $0–$19,999 range (41.4%), the $20,000–$49,999 range (29.7%), or the $50,000–$74,999 range (9.6%). 5.7% of participants had personal incomes over $100,000, and 7.8% either indicated prefer not to answer or did not answer the item. Using the range from 1 ($0–$19,999) to 9 ($300,000 or more), the mean was 2.04 (*SD* = 1.44). Regarding household income, most participants were either in the $0–$19,999 range (25.0%), the $20,000–$49,999 range (28.7%), or the $50,000–$74,999 range (16.0%). 14.0% of participants had household incomes over $100,000, and 7.0% either indicated prefer not to answer or did not answer the item. Household income ranged from 1 ($0–$19,999) to 9 ($300,000 or more), with a mean of 2.74 (*SD* = 1.81).

As shown in Table 1, responses to the stigma items varied, with the highest percentage (60%) of the sample strongly or somewhat agreeing to the item “*Most people believe that a person who does not attend 4-year college is uneducated*.” and the lowest percentage (23%) of the sample agreeing to the item “*My friends treated me badly because I did not go to 4-year college.*” The overall mean for the stigma items was 2.21 (*SD* = 0.74; range 1–4), indicating that most participants reported somewhat disagreeing to the items. With regarding to education-based discrimination, most of the sample reported having experienced discrimination based on their level of education; 71.8% of participants endorsed experiencing at least one of the four items at least once in the past year. The overall mean for the discrimination items was 2.89 (SD = 1.61; range 1–6), indicating that on average, participants experienced education-related discrimination approximately a few times a year. The item with the highest mean was “*People act as if they are better than you*” (*M* = 3.18, *SD* = 1.86), indicating that on average, participants experienced this form of discrimination a little more than a few times a year.

Next, we examined the correlation between demographic characteristics and perceptions of education-related stigma and discrimination. Age was significantly negatively associated with stigma (*r*(485) = − 0.09, *p* < 0.05) and discrimination (*r*(486) = − 0.13, *p* < 0.01). Gender identity and race were both not significantly related to education-related stigma (*F*(5, 479) = 2.17, *p* = 0.06; *F*(6, 478) = 0.97, *p* = 0.44) and education-related discrimination (*F*(5, 480) = 1.65, *p* = 0.15; *F*(6, 479) = 1.39, *p* = 0.21), respectively.^1^ Lastly, both personal and household income were not significantly related to stigma (*r*(447) = 0.02, *p* = 0.66; *r*(451) = − 0.01, *p* = 0.78) and discrimination due to education level (*r*(448) = 0.07, *p* = 0.15; *r*(452) = − 0.01, *p* = 0.77), respectively.

Because age was the only demographic variable that was associated with education-related stigma and discrimination, all four regression analyses controlled for age. As shown in Table 2, in the first regression analysis, higher mean levels of education-related stigma was associated with greater past-month anxiety symptoms. In the second analysis, higher mean levels of education-related stigma was associated with greater depression symptoms. In the third and fourth regression analyses, more frequent experiences of education-related discrimination was associated with greater past-month anxiety and depression symptoms (see Table 2)*.*

**Table 2:** Regression analyses for examining the association between stigma and discrimination on mental health symptoms

	Unstandardized coefficients		Standardized coefficients
	*B*	*SE*	Beta
Model 1: *Outcome* anxiety symptoms			
Age	0.026	0.021	0.054
Mean-level stigma	0.419	0.063	0.293***
Model 2: *Outcome* depression symptoms			
Age	0.030	0.021	0.062
Mean-level stigma	0.393	0.063	0.276***
Model 3: *Outcome* anxiety symptoms			
Age	0.031	0.021	0.065
Mean-level discrimination	0.187	0.029	0.286***
Model 4: *Outcome* depression symptoms			
Age	0.039	0.021	0.082
Mean-level discrimination	0.224	0.028	0.343***

^*^p < .05; **p < .01; ***p < .001 (2-tailed)

## Discussion

The current study is one of the first to examine education-related stigma and discrimination in a sample of emerging adults who never attended 4-year college. We found that about half of participants agreed that individuals who do not attend 4-year college are treated worse than those who do attend and are viewed as uneducated. While most participants did not think they were treated badly by friends and family members because they did not attend 4-year college, a little over half of participants felt that they had less access to resources. Furthermore, many individuals felt that they were discriminated against because they did not attend 4-year college. Taken together, these findings suggest that young adults who do not attend 4-year college experience education-related stigma and discrimination.

We found that the only demographic variable that was related to stigma and discrimination was age, such that age was inversely related to perceived stigma and discrimination. We posit that this arises because 18–19-year-olds are currently deciding on whether to attend 4-year college. Opportunities to continue education—and the potential social consequences of not continuing—may be very salient during this time. We also found that education-related stigma from parents and friends was not frequently endorsed, indicating that stigma and discrimination may come from peers or strangers. Furthermore, many individuals may be experiencing this type of discrimination and stigma when they head directly into the workforce, where the types of jobs they can attain are directly related to education. Surprisingly, we found that both household and personal income were unrelated to education-related stigma and discrimination, suggesting that this is a unique type of stigma and discrimination that is not associated with income.

Lastly, we found that experiences of education-related stigma and discrimination were related to higher depression and anxiety symptom severity. This is consistent with other research that has found that other forms of stigma and discrimination such as those that are race-related and sexual-orientated related are associated with worse physical and mental health outcomes [30, 31]. Thus, education-related stigma and discrimination may partially account for the consistently strong association between lower levels of education and worse health.

### Limitations and future directions

The current study is not without limitations. First, the sample is a non-representative convenience sample, so the generalizability of the findings is a concern. The prevalence of education-related stigma and discrimination may not be generalizable to emerging adults across the United Status. Future research should examine if the prevalence rates of education-related stigma and discrimination differ based on population size (e.g., urban versus rural) and geographic location (e.g., the South versus the Northeast). Second, the current study utilized self-report measures, so individuals may have either exaggerated or underreported their experiences of stigma and discrimination as well as symptoms of mental health. Third, the sample is cross-sectional, so we are unable to determine causality. Future research should examine how experiences of education-related stigma and discrimination are related to mental health over time. Still despite these limitations, the current study is an important first step and will hopefully stimulate interest in this area for further research.

## Conclusions

These findings could serve to increase awareness regarding the unique and significant discrimination that young adults who do not attend 4-year college experience as well as identify specific areas of intervention that can help these young adults cope with the effects of education-based stigma and discrimination. Education-related interventions may be useful in both high schools and in the general public to reduce the negative bias towards young adults who do not attend 4-year college. These interventions should identify the positive attributes of people pursing career tracks and life goals other than 4-year college. Although attending college is a positive event, individuals who do not attend college should not be viewed or treated as less than those who do attend 4-year college. These global interventions need to inform the general public that not going to 4-year college is acceptable and that these individuals can have healthy, productive, and positive lives without attending 4-year college. The findings from this study are especially relevant, given that the majority of young adults do not graduate from 4-year college [15].

## Data Availability

The raw data, syntax, and materials used in this study are not openly available but are available upon request to the corresponding author.

## Footnotes

1 Analyses were also conducted using a 3-category measure of gender identity [1. Man (cis- or trans-), 2. Woman (cis- or trans-), 3. Other], sex assigned at birth (male vs female), Hispanic ethnicity (yes vs no), and a combination of race and ethnicity (those who are Non-Hispanic White vs others). No significant differences were found.
